# Establishment, validation and evaluation of predictive model for early relapse after R0 resection in hepatocellular carcinoma patients with microvascular invasion

**DOI:** 10.1186/s12967-021-02940-0

**Published:** 2021-07-06

**Authors:** Kai Zhang, Changcheng Tao, Tana Siqin, Jianxiong Wu, Weiqi Rong

**Affiliations:** Department of Hepatobiliary Surgery, National Cancer Center/National Clinical Research Center for Cancer/Cancer Hospital, Chinese Academy of Medical Sciences (CAMS) and Peking Union Medical College (PUMC), No. 17 Panjiayuannanli, Chaoyang District, Beijing, 100021 China

**Keywords:** Hepatocellular carcinoma, Microvascular invasion, Nomogram, Early-relapse, R0 resection

## Abstract

**Backgrounds:**

This is the first study to build and evaluate a predictive model for early relapse after R0 resection in hepatocellular carcinoma (HCC) patients with microvascular invasion (MVI).

**Methods:**

The consecutive HCC patients with MVI who underwent hepatectomy in Cancer Hospital of Chinese Academy of Medical Science from Jan 2014 to June 2019 were retrospectively enrolled and randomly allocated into a derivation (N = 286) and validation cohort (N = 120) in a ratio of 7:3. Cox regression and Logistic regression analyses were performed and a predictive model for postoperative early-relapse were developed.

**Results:**

A total of 406 HCC patients with MVI were included in our work. Preoperative blood alpha-fetoprotein (AFP) level, hepatitis B e antigen (HBeAg) status, MVI classification, largest tumor diameter, the status of serosal invasion, number of tumors, and the status of satellite nodules were incorporated to construct a model. The concordance index (C-index) was 0.737 and 0.736 in the derivation and validation cohort, respectively. The calibration curves showed a good agreement between actual observation and nomogram prediction. The C-index of the nomogram was obviously higher than those of the two traditional HCC staging systems.

**Conclusion:**

We have developed and validated a prediction model for postoperative early-relapse in HCC patient with MVI after R0 resection.

**Supplementary Information:**

The online version contains supplementary material available at 10.1186/s12967-021-02940-0.

## Introduction

Hepatocellular carcinoma (HCC) is the sixth and fourth most common malignant tumor worldwide and in China, respectively. It is also the fourth and third most common cause of cancer-related death worldwide and in China, respectively [1]. The risk factors for primary HCC tumorigenesis include hepatitis B virus (HBV) infection, hepatitis C virus (HCV) infection, alcoholism, and nonalcoholic fatty liver disease cirrhosis [2]. In China, HCC is mainly caused by HBV infection, which also impacts on the prognosis of HCC patients. It has been documented that hepatitis B e antigen (HBeAg) positivity is an independent factor for poor prognosis of patients with HBV-related HCC [3]. The use of appropriate management strategies for HBV infection could slow down the development of liver cirrhosis and decrease the risk of the postoperative recurrence of HBV-related HCC [4].

It is well known that liver transplantation, radiotherapy, ablation, and hepatectomy are the main local treatment strategies for primary HCC patients. Liver transplantation has not been extensively used for the treatment of liver cancer due to the limited donor pool and high cost. With the advancements in radiotherapy technologies, HCC patients achieve a good disease control after stereotactic body radiation therapy (SBRT) treatment [5]. However, the radiotherapy is only recommended for patients with unresectable HCC who are ineligible for liver transplantation [6, 7]. The treatment efficacy of ablation therapy is commonly comparable with that of surgical resection in HCC patients [8]. However, some studies have revealed that the relapse-free survival (RFS) and overall survival (OS) in patients with a HCC diameter < 2 cm after receiving ablation therapy are shorter than those receiving surgical resection [9]. In addition, HCC patients with MVI who receive ablation therapy exhibits a higher early-recurrence rate than those receive surgical resection [10]. Therefore, surgical resection is still the main treatment strategy for radical intent in HCC patients.

Although surgery is currently the main local treatment strategy for HCC, its long-term efficacy is unsatisfactory. As reported by a literature, 2-year relapse rate reaches 54% in patients receiving surgery [11]. Dozens of studies show that predictive models for relapse in HCC patients after the radical surgery have been constructed to guide postoperative adjuvant treatment decision [12–14]. Microvascular invasion (MVI) is an independent risk factor for postoperative relapse in all reported predictive models. As reported by previous studies, the incidence rate of MVI confirmed through postoperative pathological examination is 11–60% [15] and it is 39% in our hospital [16]. According to the Standard for Diagnosis and Treatment of Primary Liver Cancer [17], MVI is a mass of cancer cells in vascular cavity with adhesion to endothelial cell observed under a microscope, which mainly occurs in portal vein. It is graded according to the count of cancer cells and the distance of MVI to tumor. The emergence of MVI impacts on the OS, and it is also a risk factor for postoperative early-relapse in HCC patients [18]. The prognosis of HCC patients with postoperative early-relapse is worse than that of patients with late-relapse [19]. Therefore, it is an unmet need to identify HCC patients who are more prone to experience postoperative early-relapse. As previously mentioned, predictive models for postoperative early-relapse in HCC patients have been established, however, a predictive model for HCC patients with MVI who are more prone to experience early-relapse has not yet been reported.

In our study, a predictive model for early-relapse in HCC patients with MVI after receiving R0 resection confirmed by postoperative pathological examination was constructed and its diagnostic performance were validated in a validation cohort.

## Methods

### Patient selection

The consecutive patients who underwent hepatectomy for HCC in Cancer Hospital of Chinese Academy of Medical Science from Jan 2014 to June 2019 were enrolled. The patients were classified into M1 and M2 subgroups on the basis of MVI status according to the Guideline for Standardized Pathological Diagnosis of Primary Liver Cancer [20]. MX defined as the emergence of MVI with unknown MVI classification. HCC patients with MVI were selected for subsequent analyses as following criteria. Inclusion criteria: (1) HCC with MVI confirmed by the postoperative pathological examination; (2) R0 resection confirmed by the postoperative pathological examination; (3) Child–Pugh A liver function prior to surgery; (4) No serious dysfunction of heart, lung or kidney that impacts on prognosis; (5) Eastern Cooperative Oncology Group (ECOG) performance status of 0–1. Exclusion criteria: (1) Death within one month following surgery; (2) No relapse and a follow-up of < 12 months; (3) Unknown status of relapse; (4) With lymph node metastasis confirmed by postoperative pathological examination. Flow chart for patient screening was shown in Additional file 1: Fig. S1.

RFS was defined as the time from the end of surgery to the relapse/metastasis or the death of any cause. According to the previous studies [11, 21] and the rules of clinical follow-up, the patients with a relapse or death within 12 months after surgery were allocated into the early-relapse group and those with a relapse or death over more than 12 months postoperatively were allocated into the late-relapse group [19]. The patients without a relapse or death until the follow-up endpoint was also allocated into the late-relapse group.

### Statistical analysis

All enrolled patients were randomly allocated into a derivation cohort (286 patients) and validation cohort in a ratio of 7:3 (120 patients). Variables independently associated with postoperative early-relapse were determined to construct a predictive model in the derivation cohort as following. First step, all clinicopathological variables collected in the present work were included in the univariate Cox model to identify risk factors significantly associated with RFS. Second step, variables identified in the first step were included in the multivariate Cox model to screen independent risk factors associated with RFS. Third step, variables identified in the second step were included in the multivariate Logistic model to determine the independent risk factors associated with postoperative early-relapse. Nomogram and web calculator were subsequently performed to established a predictive model for postoperative early-relapse in the derivation cohort.

Model discrimination measured by concordance index (C-index), model calibration measured by calibration plots and clinical practicability of the predictive model measured by decision curve analyses (DCA) were used to evaluate the predictive performance of the nomogram between patients with and without early-relapse in the derivation and validation cohorts. The predictive performance of the constructed model was compared with that of the 8^th^ edition American Joint Committee on Cancer (AJCC) staging system and Barcelona Clinic Liver Cancer (BCLC) staging system in discrimination, calibration, and clinical usefulness.

The optimal cutoff value of the nomogram was determined by maximizing the Youden index based on receiver operating characteristic (ROC) curves. The optimal cutoff value of AJCC and BCLC staging system was also determined based on ROC curves. Sensitivity, specificity, and accuracy of the predictive model, AJCC and BCLC staging system were calculated in the derivation and validation cohorts, respectively.

Categorical data were analyzed using Chi-square test or Fisher exact test. The continuous variables in a normal distribution were analyzed using *t*-test or variance analysis. The variables in a non-normal distribution were analyzed using rank sum test. A P-value less than 0.05 was considered to be statistically significant. Unless otherwise stipulated, test power (α) for multivariate analysis was set as 0.05. EmpowerStats (http://www.empowerstats.com, X&Y Solutions Inc., Boston. MA) and R software (Version 3.6.2) were used for statistical analysis and chart plots. All independent variables were screened by collinearity analysis with variance inflation factor (VIF) ≤ 5.

## Results

### Baseline characteristics of patients in the derivation and validation cohort

A total of 1320 HCC patients were retrospectively recruited in our work, including 482 patients with MVI (36.5%). According to the inclusion and exclusion criteria, a total of 406 HCC patients with MVI were selected for subsequent analyses, including 160 patients (39.4%) having a postoperative early-relapse, 67 patients (16.5%) having a postoperative late-relapse and 179 patients (44.1%) without a relapse until the end of follow-up period. One hundred and sixty (39.4%) and 246 patients (60.6%) were with early-relapse and non-early-relapse, respectively.

There were 286 patients and 120 patients were randomly allocated into the derivation and validation cohort in a ratio of 7:3, respectively, by using R language caret package with a seed of 2,020,090,873. The majority of clinical characteristics were not statistically different between the derivation and validation cohort, as shown in Table 1. The derivation cohort showed marginally higher proportion of patients with albumin-bilirubin (ALBI) score ≤ − 2.60 compared with the validation cohort (P = 0.084, Table 1). Preoperative albumin (ALB) level was marginally higher in the derivation cohort than that in the validation cohort (P = 0.077, Table 1). Preoperative alanine aminotransferase (ALT) level was significantly higher in the derivation cohort than that in the validation cohort (P = 0.021, Table 1).

**Table 1:** The clinical characteristics of patients in the derivation and validation cohort

Clinical characteristics	Derivation cohort	Validation cohort	P-value
Number of patients	286	120	
Gender			0.242
Male	237 (82.87%)	105 (87.50%)	
Female	49 (17.13%)	15 (12.50%)	
Age (years)			0.458
≤ 60	199 (69.58%)	79 (65.83%)	
> 60	87 (30.42%)	41 (34.17%)	
Hypertension			0.233
With	74 (25.87%)	38 (31.67%)	
Without	212 (74.13%)	82 (68.33%)	
Diabetes			0.885
With	46 (16.08%)	20 (16.67%)	
Without	240 (83.92%)	100 (83.33%)	
Coronary heart disease			0.803
With	11 (3.85%)	4 (3.33%)	
Without	275 (96.15%)	116 (96.67%)	
Smoking history			0.393
With	125 (43.71%)	58 (48.33%)	
Without	161 (56.29%)	62 (51.67%)	
Drinking history			0.187
With	88 (30.77%)	45 (37.50%)	
Without	198 (69.23%)	75 (62.50%)	
Portal hypertension			0.325
With	65 (22.73%)	22 (18.33%)	
Without	221 (77.27%)	98 (81.67%)	
ALBI core			0.084
≤ − 2.60	256 (89.51%)	100 (83.33%)	
> − 2.60 to ≤ − 1.39	30 (10.49%)	20 (16.67%)	
Preoperative ALT level (U/L)	30.0 (13.0–241.0)	26.50 (13.00–337.0)	0.021
Preoperative AST level (U/L)	30.0 (13.0–241.0)	26.50 (13.00–337.0)	0.104
Preoperative ALB level (g/L)	44.02 ± 4.19	43.20 ± 4.41	0.077
Preoperative serum creatinine level (mg/dl)	73.35 ± 14.29	76.21 ± 13.28	0.061
Preoperative blood glucose level (mmol/L)	5.62 ± 1.72	5.50 ± 1.37	0.489
Preoperative PT (second)	11.92 ± 0.95	11.97 ± 1.07	0.688
Preoperative status of HBsAg			0.165
Positive	232 (81.12%)	90 (75.00%)	
Negative	54 (18.88%)	30 (25.00%)	
Preoperative status of HBeAg			0.240
Positive	65 (22.73%)	21 (17.50%)	
Negative	221 (77.27%)	99 (82.50%)	
HCV-Ab			0.669
Positive	20 (6.99%)	7 (5.83%)	
Negative	266 (93.01%)	113 (94.17%)	
Preoperative LnAFP level	4.62 ± 2.95	4.62 ± 2.95	0.932
Maximum diameter of primary tumor (cm)			0.836
≤ 5	170 (59.44%)	70 (58.33%)	
> 5	116 (40.56%)	50 (41.67%)	
Adjacent to large blood vessels			0.770
With	90 (31.47%)	36 (30.00%)	
Without	196 (68.53%)	84 (70.00%)	
Adjacent to the diaphragm			0.078
With	24 (8.39%)	17 (14.17%)	
Without	262 (91.61%)	103 (85.83%)	
MVI classification			0.263
M1	132 (46.15%)	58 (48.33%)	
M2	66 (23.08%)	34 (28.33%)	
MX	88 (30.77%)	28 (23.33%)	
Number of tumors			0.326
1	250 (87.41%)	109 (90.83%)	
≥ 2	36 (12.59%)	11 (9.17%)	
Satellite nodule			0.749
With	49 (17.13%)	19 (15.83%)	
Without	237 (82.87%)	101 (84.17%)	
Serosal invasion			0.657
With	172 (60.14%)	75 (62.50%)	
Without	114 (39.86%)	45 (37.50%)	
Preoperative/intraoperative ablation			1.000
With	7 (2.45%)	2 (1.67%)	
Without	279 (97.55%)	118 (98.33%)	
Preoperative radiotherapy			0.727
With	6 (2.10%)	3 (2.50%)	
Without	280 (97.90%)	117 (97.50%)	
Preoperative interventional therapy			0.924
With	16 (5.59%)	7 (5.83%)	
Without	270 (94.41%)	113 (94.17%)	
Postoperative radiotherapy			0.642
With	47 (16.43%)	22 (18.33%)	
Without	239 (83.57%)	98 (81.67%)	
Postoperative interventional therapy			0.447
With	114 (39.86%)	43 (35.83%)	
Without	172 (60.14%)	77 (64.17%)	
AJCC staging system (the 8th edition)			0.362
Stage I	21 (7.34%)	10 (8.33%)	
Stage II	202 (70.63%)	91 (75.83%)	
Stage III	63 (22.03%)	19 (15.83%)	
BCLC staging system			0.588
Stage 0	21 (7.34%)	10 (8.33%)	
Stage A	39 (13.64%)	14 (11.67%)	
Stage B	199 (69.58%)	89 (74.17%)	
Stage C	27 (9.44%)	7 (5.83%)	
Early-relapse			0.610
Presence	115 (40.21%)	45 (37.50%)	
Absence	171 (59.79%)	75 (62.50%)	

*ALBI* albumin-bilirubin, *ALT* alanine aminotransferase, *AST* aspartate aminotransferase, *ALB* albumin, *PT* prothrombin time, *HBsAg* hepatitis B surface antigen, *HBeAg* hepatitis B e antigen, *HCV-Ab* hepatitis C virus-antibody, *MVI* microvascular invasion, *AFP* alpha-fetoprotein, *AJCC* American Joint Committee on Cancer, *BCLC* Barcelona Clinic Liver Cancer

### Determination of risk factors associated with postoperative early-relapse

Next, the univariate and multivariate Cox regression analyses were performed to determine the independent risk factors associated with RFS in the derivation cohort. Total of 6 independent risk factors associated with RFS were identified, including ALBI score, preoperative HBeAg status, MVI classification, largest tumor diameter, number of tumors and the status of serosal invasion (Table 2). Preoperative blood AFP level and the status  of satellite nodule were marginally statistically associated with RFS. As previously reported, blood AFP level is significantly associated with prognosis and early-relapse, and the multinodular tumor commonly develops from MVI in HCC patients [20], therefore, these two variables were further incorporated into the subsequent analysis. Next, the abovementioned 8 variables were included in the multivariable Logistic regression analysis to determine the risk factors associated with postoperative early-relapse. Total of 5 independent risk factors were identified, including preoperative blood AFP level, preoperative HBeAg status, MVI classification, largest tumor diameter and the status of serosal invasion (Table 2). Number of tumors and the status of satellite nodules were marginally statistically associated with early-relapse (Table 2). The previous studies have revealed that both number of tumors and the status of satellite nodules are independent risk factors for postoperative relapse [18, 22], therefore, these two variables were included for the subsequent analysis to establish a predictive model.

**Table 2:** Univariable and multivariable Cox analyses of risk factors for RFS and multivariable Logistic analysis of risk factors for early-relapse in HCC patients with MVI who underwent R0 resection

Variables	No./mean ± SD	Univariate cox analysis			Multivariate cox analysis			Multivariate logistic analysis		
		HR	95%CI	P-value	HR	95%CI	P-value	OR	95%CI	P-value
Gender										
Male	237 (82.87%)	reference								
Female	49 (17.13%)	0.87	0.56–1.34	0.519						
Age (years)										
≤ 60	199 (69.58%)	reference								
> 60	87 (30.42%)	0.89	0.63–1.24	0.479						
Hypertension										
Without	212 (74.13%)	reference								
With	74 (25.87%)	1.23	0.88–1.73	0.2305						
Diabetes										
Without	240 (83.92%)	reference								
With	46 (16.08%)	0.96	0.63–1.46	0.8547						
Coronary heart disease										
Without	275 (96.15%)	reference								
With	11 (3.85%)	1.15	0.54–2.46	0.7171						
Smoking history										
Without	161 (56.29%)	reference								
With	125 (43.71%)	1.05	0.77–1.43	0.7508						
Drinking history										
Without	198 (69.23%)	reference								
With	88 (30.77%)	0.96	0.69–1.33	0.802						
Portal hypertension										
Without	221 (77.27%)	reference								
With	65 (22.73%)	1.27	0.89–1.81	0.1855						
Preoperative Ln AFP level	4.62 ± 2.95	1.07	1.02–1.13	**0.0081**	1.05	1.00–1.11	**0.063**	1.12	1.02- 1.23	**0.0147**
ALBI score										
≤ − 2.60	256 (89.51%)	reference			reference					
> − 2.60 to ≤ − 1.39	30 (10.49%)	2.15	1.41–3.27	**0.0004**	1.94	1.10–3.43	**0.0218**	2.04	0.86- 4.82	0.1056
Preoperative PT (second)	11.92 ± 0.95	1.12	0.95–1.32	0.1671						
Preoperative blood glucose level (mmol/L)	5.62 ± 1.72	1.05	0.96–1.16	0.276						
Preoperative serum creatinine level (mg/dl)	73.35 ± 14.29	0.99	0.98–1.00	0.1635						
Preoperative ALB (g/L)	44.02 ± 4.19	0.95	0.91–0.98	**0.004**	1.01	0.96–1.05	0.8346			
Preoperative ALT (U/L)	35.16 ± 23.38	1	1.00- 1.01	0.2086						
Preoperative AST (U/L)	34.68 ± 22.96	1.01	1.00- 1.01	**0.006**	1	1.00–1.01	0.5784			
Preoperative HBsAg										
Positive	232 (81.12%)	reference								
Negative	54 (18.88%)	1.08	0.74–1.58	0.692						
Preoperative HBeAg										
Negative	221 (77.27%)	reference			reference					
Positive	65 (22.73%)	1.43	1.01–2.02	**0.0454**	1.52	1.04–2.20	**0.0286**	1.93	1.02- 3.65	**0.0427**
HCV-Ab										
Negative	266 (93.01%)	reference								
Positive	20 (6.99%)	1.17	0.67–2.07	0.5795						
Adjacent to large blood vessels										
Without	196 (68.53%)	reference								
With	90 (31.47%)	1.17	0.84–1.61	0.3517						
Adjacent to the diaphragm										
Without	262 (91.61%)	reference								
With	24 (8.39%)	2.14	1.34–3.42	**0.0014**	1.24	0.74–2.09	0.4138			
MVI classification										
M1	132 (46.15%)	reference			reference			reference		
M2	66 (23.08%)	2.7	1.83–3.99	** < 0.0001**	2.14	1.41–3.25	**0.0004**	2.3	1.17- 4.52	**0.0157**
MX	88 (30.77%)	1.7	1.17–2.47	**0.0054**	1.29	0.86–1.93	0.21	1.65	0.88- 3.12	0.1202
Largest tumor diameter (cm)										
≤ 5	170 (59.44%)	reference			reference					
> 5	116 (40.56%)	1.99	1.46–2.71	** < 0.0001**	1.56	1.10–2.22	**0.0124**	1.78	1.01- 3.14	**0.0476**
Number of tumors										
1	250 (87.41%)	reference			reference			reference		
≥ 2	36 (12.59%)	1.77	1.17–2.66	**0.0064**	1.66	1.08–2.55	**0.0209**	2.06	0.93- 4.56	**0.0755**
Satellite nodule										
Without	237 (82.87%)	reference		reference				reference		
With	49 (17.13%)	1.79	1.23–2.59	**0.0022**	1.44	0.97–2.14	**0.0726**	1.95	0.97- 3.91	**0.0606**
Serosal invasion										
Without	114 (39.86%)	reference		reference				reference		
With	172 (60.14%)	1.97	1.41–2.76	** < 0.0001**	1.59	1.11–2.28	**0.0109**	2.06	1.17- 3.64	**0.0129**
Preoperative/intraoperative ablation										
Without	279 (97.55%)	reference								
With	7 (2.45%)	1.07	0.34–3.35	0.9105						
Preoperative radiotherapy										
Without	280 (97.90%)	reference								
With	6 (2.10%)	1.08	0.34–3.38	0.9000						
Preoperative interventional therapy										
Without	270 (94.41%)	reference								
With	16 (5.59%)	1.56	0.85–2.88	0.1536						
Postoperative radiotherapy										
Without	239 (83.57%)	reference								
With	47 (16.43%)	0.89	0.59–1.35	0.5919						
Postoperative interventional therapy										
Without	172 (60.14%)	reference								
With	114 (39.86%)	1.23	0.90–1.67	0.1919						

*HCC *hepatocellular carcinoma, *MVI* microvascular invasion, *No.* number, *SD* standard deviation, *ALBI* albumin-bilirubin, *ALT* alanine aminotransferase, *AST* aspartate aminotransferase, *ALB* albumin, *PT* prothrombin time, *HBsAg* hepatitis B surface antigen, *HBeAg* hepatitis B e antigen, *HCV-Ab* hepatitis C virus-antibody, *MVI* microvascular invasion, *AFP* alpha-fetoprotein, *AJCC* American Joint Committee on Cancer, *BCLC* Barcelona Clinic Liver Cancer, *HR* hazard ratio, *CI* confidence interval, *OR* odds ratio

### Establishment of predictive model for postoperative early-relapse and evaluation of its discriminability and calibration

**Fig. 1:**
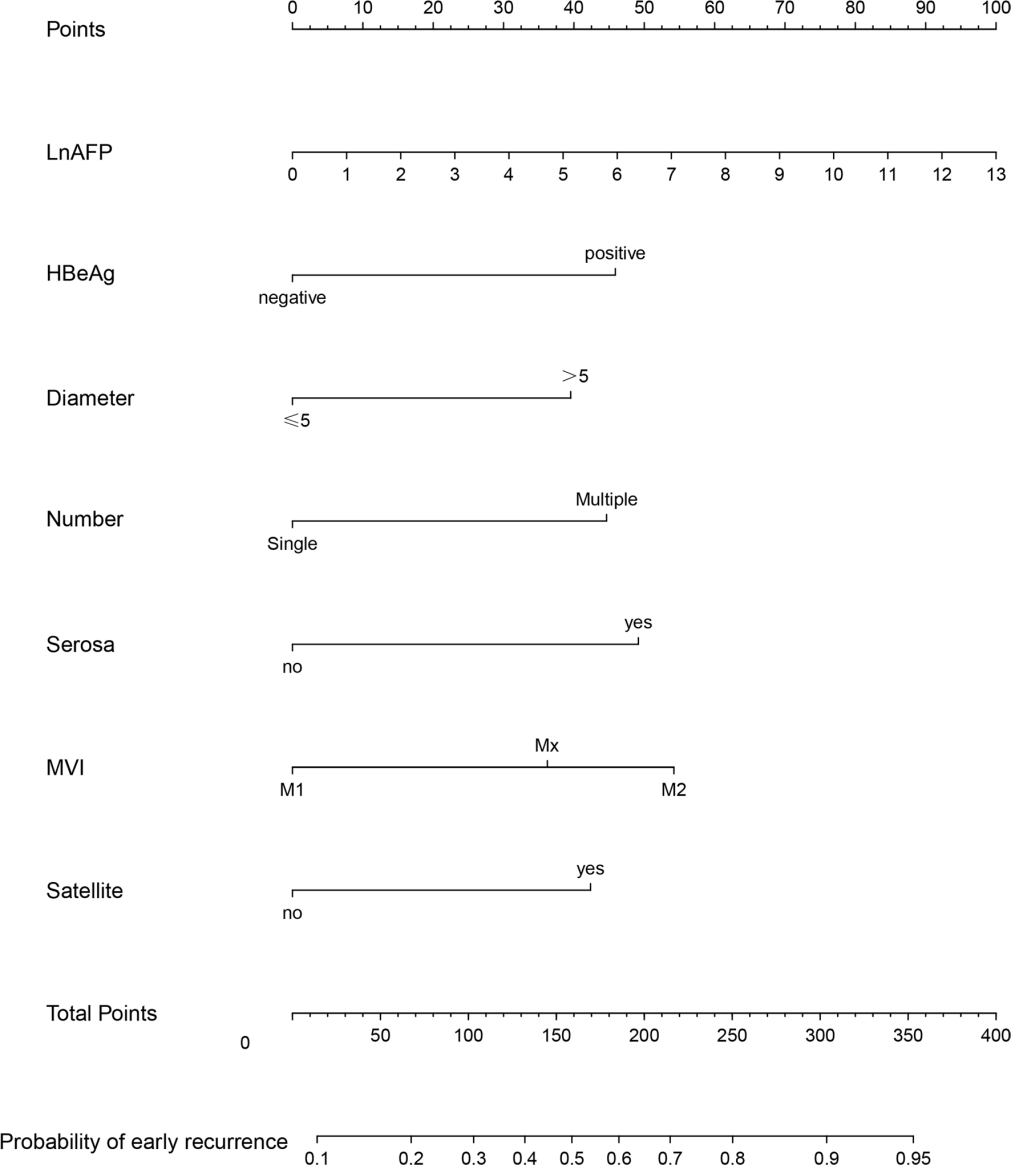
Nomogram in HCC patients with MVI after R0 resection. *HCC* hepatocellular carcinoma, *MVI* microvascular invasion

Next, the abovementioned 7 risk factors associated with postoperative early-relapse were included to construct the predictive model by using a binary logistic regression equation and the results were displayed in nomogram (Fig. 1). We also provided a web calculator at the website (https://zhangkaimedicalapp.shinyapps.io/DynNomapp/) for clinicians to use this model to predict the probability of postoperative early-relapse in HCC patients with MVI (Additional file 2: Fig. S2). In the derivation cohort, the C-index of discrimination was 0.737, 0.60 and 0.57 for the predictive model, AJCC and BCLC staging system, respectively. In the validation cohort, the C-index of discrimination was 0.736, 0.63 and 0.60 for the predictive model, AJCC and BCLC staging system, respectively. Collectively, the predictive model had an acceptable discriminability both in the derivation and validation cohort. The resampling was done for 1000 times using Bootstrap method to assess the model calibration. The predictive probability was consistent with the actual probability either in the derivation or validation cohort (Additional file 3: Fig. S3). Taken together, the predictive model was feasible to accurately predict postoperative early-relapse in HCC patients with MVI.

### Comparison of the predictive value of the model and traditional staging systems in diagnosis of postoperative early-relapse

**Fig. 2:**
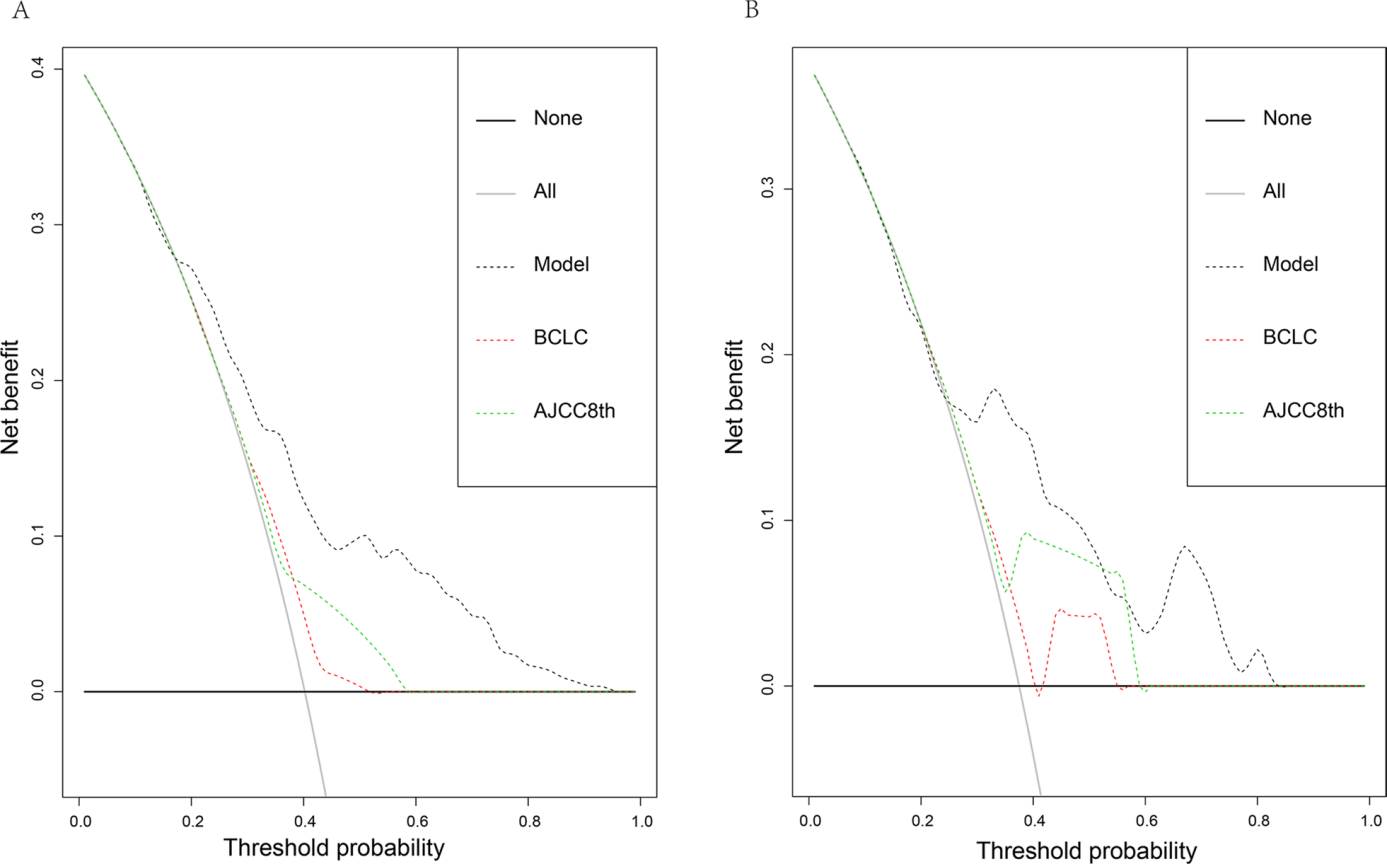
Decision curve analyses in the derivation (**A**) and validation (**B**) cohort. *AJCC* American Joint Committee on Cancer, *BCLC* Barcelona Clinic Liver Cancer

DCA curve was plotted and the predictive value of the constructed model in clinical practicability was compared with that of the 8th edition AJCC staging system and BCLC staging systems. As shown by Fig. 2, in either the derivation or validation cohort, DCA curve for the predictive model was above those for traditional staging systems, which suggested that the predictive model was superior to traditional staging systems in terms of threshold probability. In order to further explore the practicable value of the predictive model, ROC curve was plotted for the predictive model, AJCC staging system and BCLC staging system and optimal cutoff value was calculated, respectively. According to the optimal cutoff value of 120 points calculated based on the Youden index, 406 patients were allocated into high- (> 120 points) and low risk-group (< 120 points) and actual status of postoperative early-relapse were compared between the two groups. In the derivation group, the sensitivity and specificity for the predictive model were 74% and 61% (Table 3). The sensitivity and specificity were 76% and 64% for the predictive model in the validation cohort (Table 3). Although the sensitivity of BCLC staging system was superior to that of the predictive model, its specificity was only 26% and 24% in the derivation group and validation group, respectively (Table 3). The specificity of AJCC staging system was superior to that of the predictive model, however, its sensitivity was only 32% and 31% in the derivation and validation cohort, respectively (Table 3). Taken together, the predictive model had the good sensitivity and specificity either in the derivation and validating cohort.

**Table 3:** Prognostic performance of the developed model for postoperative early-relapse in HCC patients with MVI

Performance	Derivation cohort			Validation cohort		
	Predictive model	BCLC staging system	AJCC system (the 8^th^ edition)	Predictive model	BCLC staging system	AJCC system (the 8^th^ edition)
Best cutoff value	120 points	Stage A	Stage III	120 points	Stage A	Stage III
Sensitivity (%)	74	86	32	76	86	31
Specificity (%)	61	26	85	64	24	93
Accuracy (%)	66	61	64	68	47	70

*HCC* hepatocellular carcinoma, *MVI* microvascular invasion, *AJCC* American Joint Committee on Cancer, *BCLC* Barcelona Clinic Liver Cancer

## Discussion

In this study, a predictive model for postoperative early-relapse in HCC patients with MVI was established, which were displayed using nomogram and webpage calculator for convenient clinical application (https://zhangkaimedicalapp.shinyapps.io/DynNomapp/). In this study, 406 HCC patients with MVI were selected as subjects. Univariate and multivariate analyses were performed in the derivation cohort to identify risk factors associated with early-relapse, which were used to construct a model to predict the possibility of early-relapse in HCC patients with MVI. The discrimination, calibration and clinical usefulness of the predictive model were superior to that of traditional staging systems, such as AJCC and BCLC. According to the optimal cutoff value of 120 points, the patients were further allocated into the high- and low-risk group, the predictive model showed the good sensitivity and specificity in distinguishing patients with high-risk early-relapse after R0 resection from those with low-risk.

The prognosis of HCC patients is mainly influenced by the following three factors: (1) Factors of patient-self, such as status of hepatitis virus infection and liver function; (2) Factors of tumor, such as diameter of tumor, MVI classification, and blood AFP level; (3) Factors of treatment, such as postoperative adjuvant treatment. In this study, 6 of all 7 risk factors associated with early-relapse were tumor-related factors, including preoperative blood AFP level, MVI classification, number of tumors, largest tumor diameter, the status of serosal invasion and satellite nodules. These results indicate that tumor-related factors play important roles in the postoperative early-relapse of HCC patients with MVI.

It has been documented that preoperative blood AFP level, number of tumors, and diameter of tumor are risk factors for predicting prognosis of patients [12, 14, 22, 23]. Blood AFP level might be positively related to the diameter of tumor in HCC patients, and the presence of tumor enlargement is a predictive factor for poor prognosis [24]. A previous study has reported that blood AFP level is still the independent risk factor for poor prognosis after adjusting for the presence of tumor enlargement [25]. At present, blood AFP level of > 400 ng/mL is commonly considered as the independent risk factor for poor prognosis [26]. The HCC patients with multiple tumors can be classified into patients with intrahepatic metastasis and with intrahepatic multiple primary tumors [27]. Intrahepatic metastasis indicates the disease progression on primary HCC and intrahepatic multiple primary tumors are commonly related to poor liver function. Early-relapse is more prone to occur in HCC patients with intrahepatic metastasis, and late-relapse is more prone to occur in patients with poor liver function [28]. In this study, patients were not stratified according to the status of multiple tumors. The number of tumors was identified as a risk factor with a marginally statistical difference for early-relapse might be largely due to the fact that some patients with intrahepatic multiple primary tumors were incorporated into our study.

In current viewpoints, MVI and the presence of satellite nodules are different stages during the progression of tumor. The emergence of satellite nodules indicates disease progression on HCC with MVI [20]. As shown by previous studies, MVI is an important risk factor for postoperative poor prognosis in HCC patients, and it also could predict the postoperative early-relapse [18]. The patient with high grade of MVI exhibits poor prognosis [29]. The presence of multinodular tumor is regarded as resulting from disease progression of MVI, and it is related to poor prognosis of patients [28]. It is well known that tumor size is related to the prognosis of patients. The presence of tumor enlargement predicts poor prognosis of HCC patients. The corresponding cutoff value of tumor size is used in different guidelines to predict prognosis because the correlation between tumor size and poor prognosis in patients is not in a linear manner. In this study, by referring to relevant indices recommended in the Chinese Standards for Diagnosis and Treatment of Primary Liver Cancer [17], AJCC staging system (the 8^th^ edition) and Hong Kong staging system for liver cancer [30], the cutoff value was set as 5 cm in our work. Tumor with a diameter > 5 cm was also identified as an independent risk factor for early-relapse in the present work.

The previous studies have demonstrated that incomplete tumor encapsulation predicts poor prognosis in HCC patients [18, 22]. In this study, the association between tumor encapsulation and prognosis was not investigated because the status of tumor encapsulation in most of HCC patients was unknown. The status of serosal invasion was included in the study. Serosal invasion is defined as the microscopic invasion of tumor to fibrous membrane of liver. Our work indicated that the emergence of serosal invasion was the independent risk factor for early-relapse.

Postoperative adjuvant treatment, such as postoperative interventional therapy and radiotherapy, could improve the prognosis of HCC patients with MVI [31, 32]. In our study, the postoperative interventional therapy/radiotherapy was not significantly associated with the prognosis of patients, which might be attributed to the different baseline data between patients with and without treatment and the small sample size. A study from our hospital has demonstrated that RFS is longer in patients with a narrow incision margin (< 1 cm) after receiving postoperative radiotherapy compared with those receiving postoperative interventional therapy by using the propensity score matching method [33]. Another study also has revealed that postoperative radiotherapy improves the prognosis of patients with a narrow incision margin (< 1 cm) [34]. M2 MVI was defined as MVI occurring at > 1 cm away from primary tumor or the number of MVI more than 5 [20]. Therefore, the prognosis of patients with M2 MVI may be improved after the postoperative radiotherapy because it is theoretically possible for such patients to have relatively narrow surgical margins.

In this study, among the factors of patient-self, HBeAg status was associated with postoperative early-relapse in HCC patients with MVI. A previous study has shown that HBeAg positivity predicts poor survival in HCC patients who underwent hepatectomy, which might be attributed to persistent liver injury resulting from an active virus replication [35]. Portal vein hypertension is also a poor prognostic factor in HCC patients with MVI [36]. In this study, portal vein hypertension was not significantly correlated with the postoperative early-relapse. Portal vein hypertension is one of adverse outcomes of persistent liver injury and impacts on the use of target drugs (such as Sorafenib). Portal vein hypertension and adjuvant treatments might impact on the prognosis of patients. In this study, both ALBI score for assessing the liver function and preoperative/postoperative adjuvant treatment were included. ALBI score is developed based on the data of a large sample size study in Japanese patients with different stage of HCC. The previous study has shown that only bilirubin and albumin level as non-tumor-related factors impact on survival [37]. This linear predictor was calculated using the following formula: (Log_10_ bilirubin level × 0.66) + [albumin level × (− 0.085)] (the unit of bilirubin and albumin level and was μmol/L and g/L, respectively). The patients were subsequently grouped into: Grade I (≤ − 2.60 points), Grade II (> − 2.60 to ≤ − 1.39 points) and Grade III (> − 1.39 points) according to the two cutoff values. In this study, ALBI score was an independent risk factor for disease relapse in HCC patients with MVI. Similar result is also shown in a previous study [38]. However, as shown by multivariate Logistic regression analysis in this study, ALBI score was not the independent risk factor for early-relapse, which might be due to the fact that poor liver function is more prone to occur in patients with late-relapse after the surgery than those with early-relapse. Similar result is shown in the previous study indicating that poor liver function increases the risk of postoperative late-relapse [28].

This study had some limitations. This study was a single-center, retrospective, and case-controlled study, which might result in the bias of our conclusions. Although the risk factors found in this study were supported by relevant studies, their internal and external truthfulness is needed to be verified. Due to a long-time span of the study, some prognostic factors were not be collected, which might result in the bias of the final conclusions. In this study, an external validation was performed in a validation group and an internal validation was performed by resampling for 1000 times using the bootstrap method in the derivation group. However, the clinical usefulness of predictive model was not validated in a real-world study. A pragmatic randomized controlled trial is needed to determine the power of the predictive model and improve the predictive model.

## Conclusion

Our work indicated that MVI classification, HBeAg status, preoperative blood AFP level, number of tumors, largest tumor diameter, the status of satellite nodules and serosal invasion were independent risk factors for early-relapse in HCC patients with MVI after R0 resection. The predictive model established by using the abovementioned risk factors was a feasible tool to predict the possibility of early-relapse in HCC patients with MVI after R0 resection.

## Supplementary Information

**Additional file 1: Fig. S1.** Flow chart of enrolled patients. HCC, hepatocellular carcinoma; MVI, microvascular invasion.

**Additional file 2: Fig. S2.** We provided a Web calculator (https://zhangkaimedicalapp.shinyapps.io/DynNomapp/) for predicting the probability of postoperative early-relapse in HCC patients with MVI. HCC, hepatocellular carcinoma; MVI, microvascular invasion.

**Additional file 3: Fig. S3.** Calibration curves in the derivation (A) and validation (B) cohort. AJCC, American Joint Committee on Cancer; BCLC, Barcelona Clinic Liver Cancer.

## Data Availability

The datasets used and/or analyzed during the current study are available from the corresponding author on reasonable request.

## Abbreviations

HCC: Hepatocellular carcinoma
MVI: Microvascular invasion
AFP: Alpha-fetoprotein
HBeAg: Hepatitis B e antigen
HBV: Hepatitis B virus
HCV: Hepatitis C virus
SBRT: Stereotactic body radiation therapy
RFS: Relapse-free survival
OS: Overall survival
ECOG: Eastern Cooperative Oncology Group
C-index: Concordance index
AJCC: American Joint Committee on Cancer
BCLC: Barcelona Clinic Liver Cancer
ROC: Receiver operating characteristic curves
VIF: Variance inflation factor
ALBI: Albumin-bilirubin
ALB: Albumin
ALT: Alanine aminotransferase
DCA: Decision curve analyses
