# Supplementary figures and images for: Establishment, validation and evaluation of predictive model for early relapse after R0 resection in hepatocellular carcinoma patients with microvascular invasion
Source: J Transl Med. 2021 Jul 6;19:293. doi: 10.1186/s12967-021-02940-0 (PMC8261942; doi:10.1186/s12967-021-02940-0)

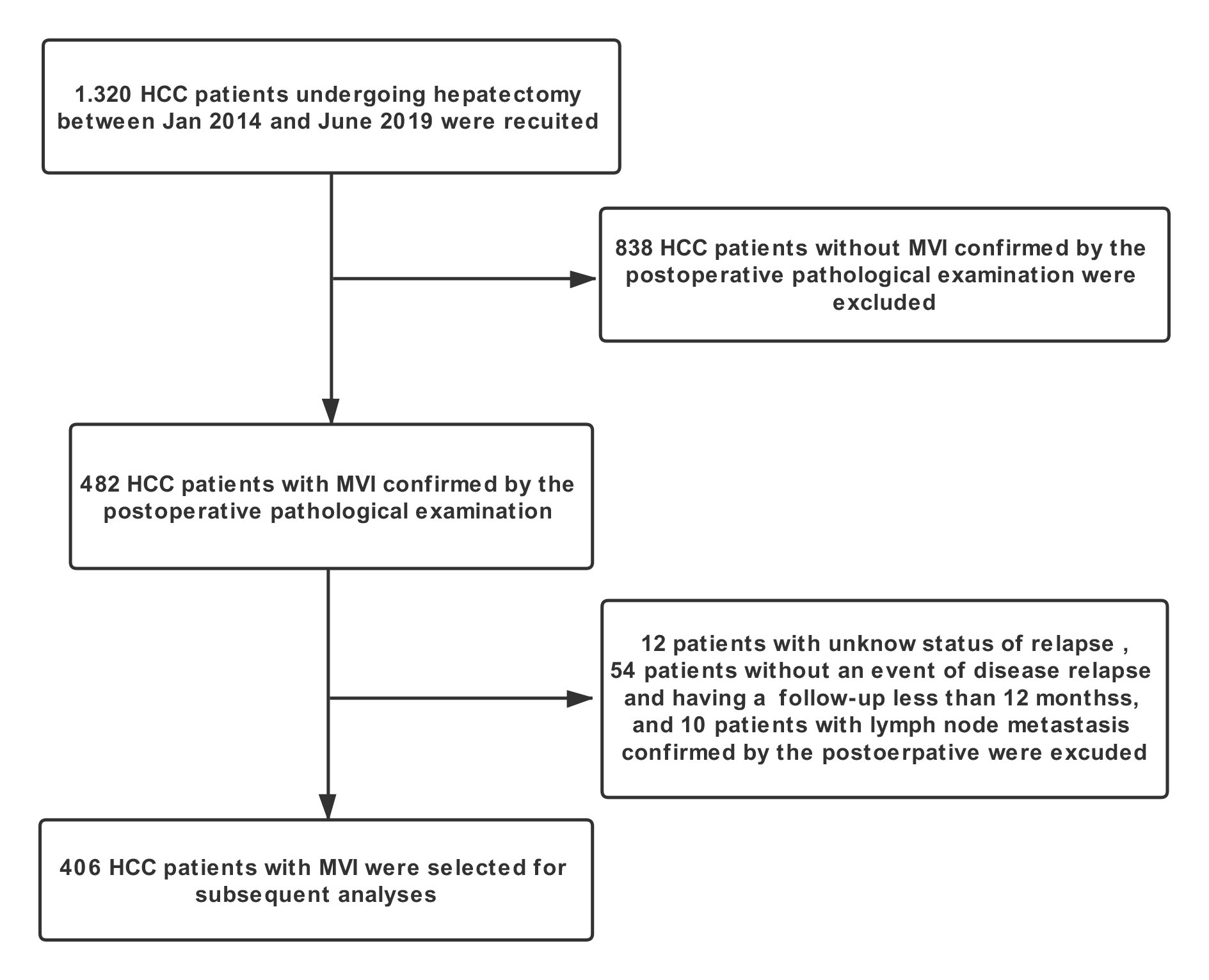

Supplement: Supplementary file 1 — Additional file 1: Fig. S1. Flow chart of enrolled patients. HCC, hepatocellular carcinoma; MVI, microvascular invasion. [file 12967_2021_2940_MOESM1_ESM.tif]

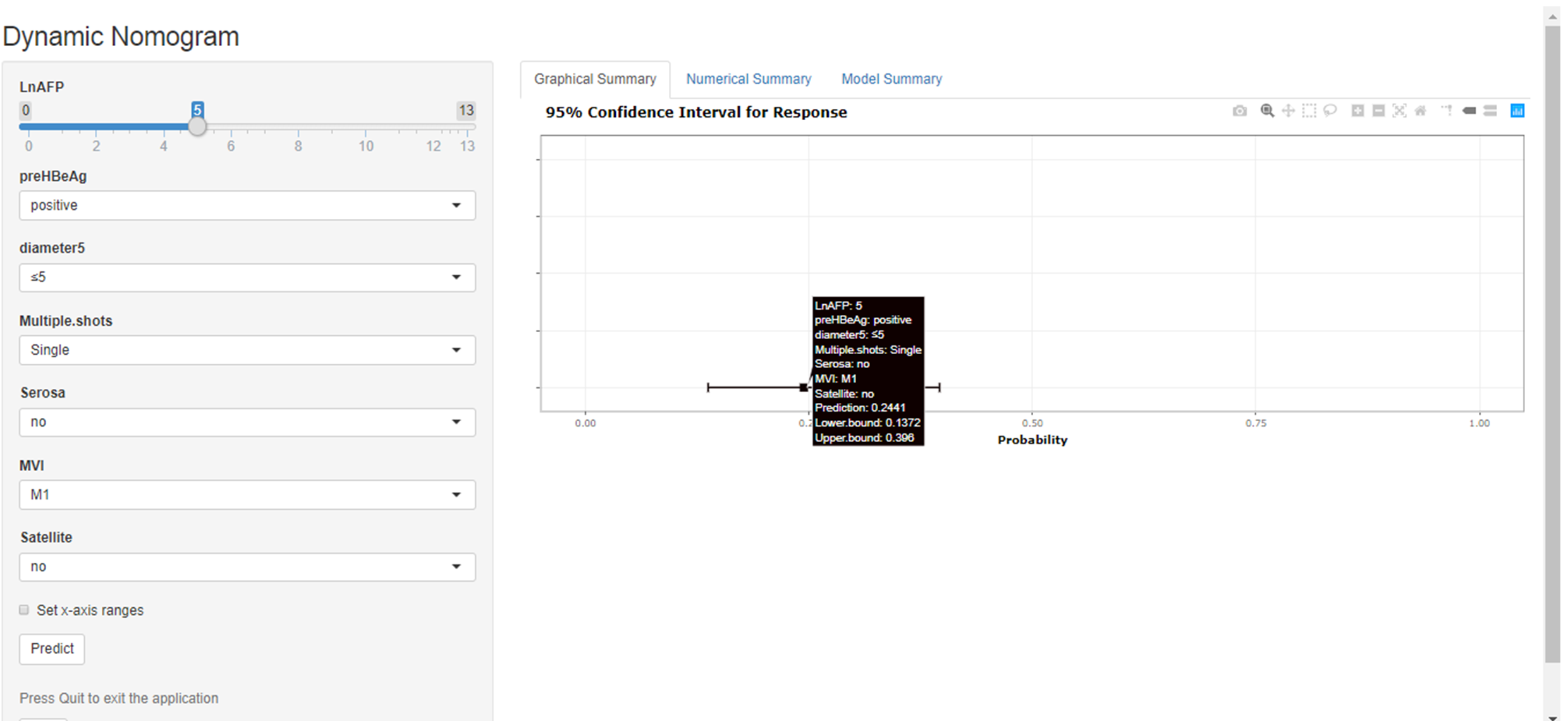

Supplement: Supplementary file 2 — Additional file 2: Fig. S2. We provided a Web calculator (https://zhangkaimedicalapp.shinyapps.io/DynNomapp/) for predicting the probability of postoperative early-relapse in HCC patients with MVI. HCC, hepatocellular carcinoma; MVI, microvascular invasion. [file 12967_2021_2940_MOESM2_ESM.tif]

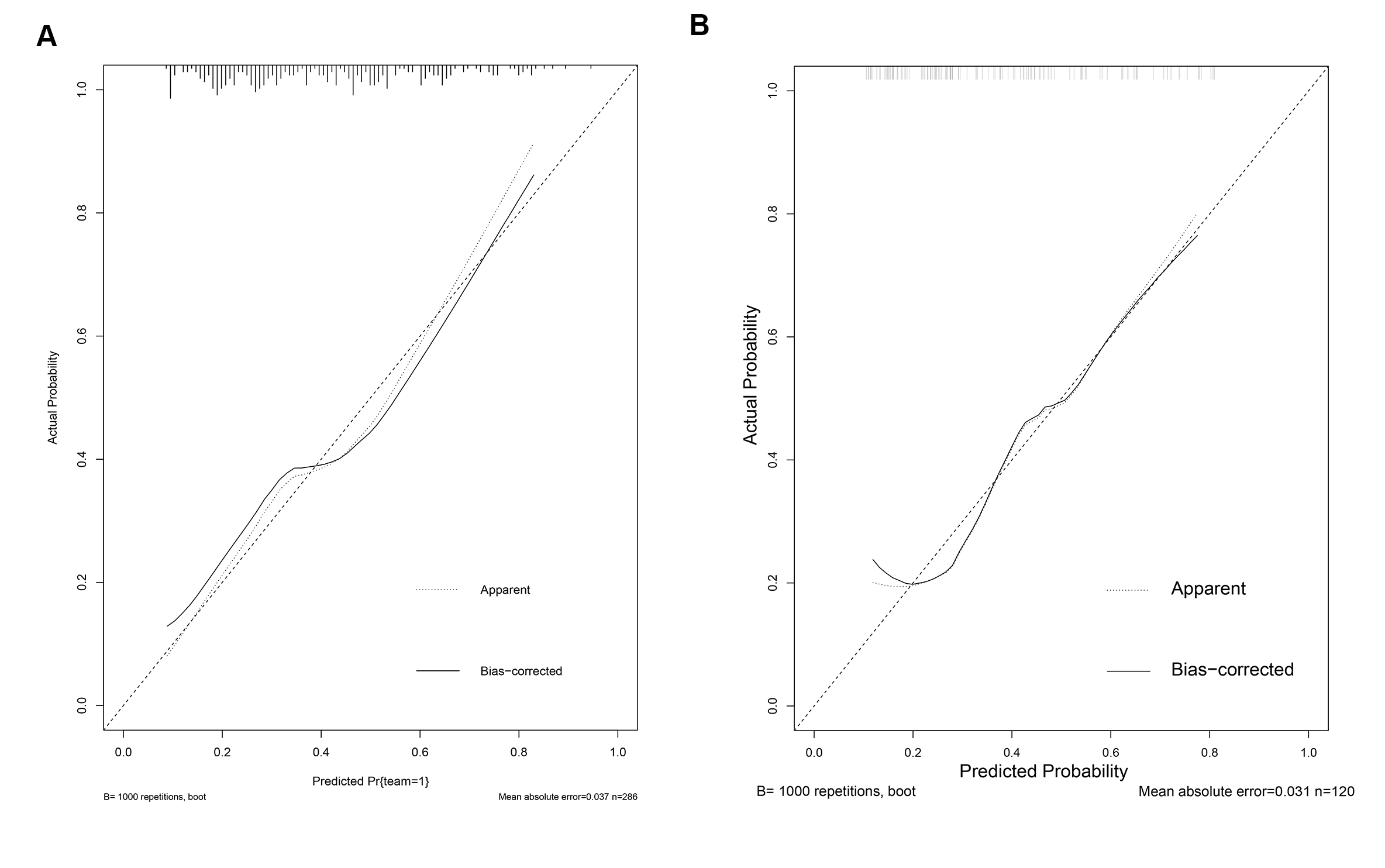

Supplement: Supplementary file 3 — Additional file 3: Fig. S3. Calibration curves in the derivation (A) and validation (B) cohort. AJCC, American Joint Committee on Cancer; BCLC, Barcelona Clinic Liver Cancer. [file 12967_2021_2940_MOESM3_ESM.tif]
